# Combined Treatment With Lipoprotein Apheresis and Hemodialysis in Patients With Severe Cardiovascular Disease, High Lipoprotein(a) and End Stage Renal Disease

**DOI:** 10.1002/jca.70020

**Published:** 2025-04-11

**Authors:** Tilmann Röseler, Daniel Kayser, Georg Schlieper, Wanja M. Bernhardt

**Affiliations:** ^1^ Center for Hypertension, Kidney‐ and Metabolic Diseases Hanover Hannover Germany; ^2^ Department of Nephrology and Hypertension University Hospital Erlangen‐Nuremberg Erlangen Germany

**Keywords:** ACVE, dialysis, lipid disorder, lipoprotein apheresis, lipoprotein(a)

## Abstract

Elevated Lipoprotein(a) (Lp(a)) is a known independent cardiovascular risk factor. Lp(a) Lipoprotein Apheresis (LA) substantially reduces the number of cardiovascular events. The effect of LA treatment in hemodialysis (HD) patients remains unknown. Retrospective analysis of nine patients undergoing LA and HD. Cardiovascular risk factors and the efficacy of treatment were assessed. Adverse cardiac or vascular events (ACVE) were recorded. Median (range) years on HD were 4.2 (1.5 to 23.6) years and median years on LA were 4.0 (1.6 to 12.4) years. Before initiation of LA, median (range) Lp(a) level was 242.67 (164.0 to 400.10) nmol/L and mean LDL‐C level (±SD) 2.49 (±1.14) mmol/L. Under treatment, mean acute reduction rates, comparing concentrations before and after LA sessions, were 64.15 (±5.45)% for Lp(a) and 57.26 (±7.93)% for LDL‐C. Before initiation of LA, 14 ACVE occurred; after initiation, only 6 (57.2% reduction rate). In this small cohort, LA appears to be effective in reducing ACVE in patients on HD with elevated Lp(a) levels. Further studies are needed to evaluate the effect of LA on cardiovascular outcomes in dialysis patients.

**Abbreviations**
ACVE: adverse cardiac or vascular events
CAD: coronary artery disease
CKD: chronic kidney disease
CVD: cardiovascular disease
eGFR: estimated glomerular filtration rate
ESRD: end‐stage renal disease
HD: hemodialysis
LA: Lipoprotein Apheresis
Lp(a): lipoprotein(a)
MACE: major adverse cardiac event
PAD: peripheral artery disease
s‐creatinine: serum‐creatinine

## 1 Introduction

Both nephrosclerosis and cardiac diseases due to severe hyperlipidemia may lead to the emergence or progression of renal disease. Vice versa, decreasing renal function leads to an increased cardiovascular risk and specific changes in the lipid profile. In end‐stage renal disease (ESRD) a number of “non‐traditional” cardiovascular risk factors, such as hyperphosphatemia, uremia, anemia, albuminuria, and specific dyslipoproteinemia, are present in addition to the well‐described traditional cardiovascular risk factors, such as hypertension, age, sex, and hypercholesterolemia [1].

According to the number of risk factors, cardiovascular morbidity and mortality of hemodialysis patients is dramatically high independent of race, age, and sex [1, 2] and it remains extremely high in CKD up to now [3]. In particular, mortality after myocardial infarction is almost 90% after 5 years [4]. In CKD, there are both quantitative changes in lipoproteins and potentially more relevant qualitative changes, specifically, an increase in small‐density‐LDL‐particles seen in LDL‐C. These small‐density‐LDL‐particles have a high propensity to penetrate vessel walls, oxidize, and thus trigger atherosclerosis [5, 6].

Lipoprotein(a) (Lp(a)), elevated in both CKD and HD [5, 6], is a very heterogeneous molecule consisting of an LDL particle, an apolipoprotein(a) and a various number of kringle IV‐repeats determining the size and molecular weight of the specific, individual Lp(a) molecule [7]. With decreasing renal function, Lp(a) has been shown to increase; however, this was only the case for high molecular weight (HMW) Lp(a) [8], whereas serum levels of low molecular weight (LMW) Lp(a) (known to be more present in patients with very high serum levels of Lp(a) and more atherogenic [9]) seem to be independent of renal function and dialysis [8].

Lp(a) has been described as an independent risk factor for cardiovascular disease in the general population [9] with the risk of occurrence of cardiovascular events increasing as plasma levels of Lp(a) increase [10]. Dialysis patients with small Apo(a) isoforms and Lp(a) levels of more than 123 nmol/L have a 73% higher risk of cardiovascular events [11], whereas the association is less clear in diabetics on HD [12].

The overall role of Lp(a) as an independent cardiovascular risk factor in CKD, in particular HD, remains unclear. The existing longitudinal retrospective studies on the role of Lp(a) in ESRD have only evaluated heterogeneous results (reviewed in [5]). Furthermore, due to the lack of specific Lp(a) lowering agents, there are no interventional studies available on these patients. Although specific Lp(a) lowering therapies may be available in the future [13], as of now, the only available treatment for elevated Lp(a) with progressive cardiovascular disease remains Lipoprotein Apheresis (LA). Lp(a)‐Apheresis is effective in reducing plasma levels of lipids, inflammatory compounds, and pro‐thrombotic proteins [14, 15]. Clinically, this has resulted in a considerable reduction in the number of major adverse cardiac events (MACE), 78% after 1 year of LA compared to the 2‐year pre‐LA period [16]. This effect is even more pronounced after 2 years of LA and remains stable for years under continued LA therapy [17, 18].

According to Kwan et al. [5], nephrologists and primary care physicians should identify and treat dyslipidemia using guidelines developed for the general population. In Germany, approval and reimbursement of LA is applicable for the isolated elevation of Lp(a) (> 60 mg/dL or 120 nmol/, respectively), in the setting of progressive cardiovascular disease [17]. Accordingly, at this study location, patients with ESRD and high levels of Lp(a) with progressive cardiovascular disease were treated with both HD and LA. This study reports findings on a series of nine patients receiving both HD and LA.

## 2 Patients and Methods

### 2.1 Patients

Retrospective analysis was completed on *n* = 9 patients undergoing combination treatment with HD and LA for more than 1 year. Data was collected from 3 years prior to initiation of LA until the reference date of the 1st of March 2021, with the 3 years prior serving as a control period. As of the designated end date, eight patients had received combined HD‐LA treatment for 3 years and five patients had received combination HD‐LA treatment for 4 years.

All other cardiovascular risk factors of patients were assessed, including sex, weight, age, time on HD, diabetes, history of smoking, arterial hypertension, and positive family history for cardiovascular events (Table 1).

**TABLE 1:** Patient characteristics at the time of analysis (1st of March 2021).

Pat.	Sex	Median age	Median years on HD	HD time per treatment (hours)	Hyper‐tension	Smoking history	Weight (kg)	Median years on LA	LA before HD	Diabetes	Indication for LA
1	f	70	9	5	1	Until 2010	60	3	0	0	Lp(a)
2	f	67	23.6	5	1	0	57.5	5.8	0	0	Lp(a)
3	m	71	4.2	5.5	1	0	109.5	3.3	0	1	Lp(a)
4	m	60	5.5	5	1	0	109	6.3	1	1	Lp(a)
5	m	80	3.9	5	1	0	78	4	1	0	LDL‐C/Lp(a)
6	f	70	1.5	5	1	0	99	5.1	1	1	Lp(a)
7	m	72	1.6	4	1	0	83.5	3.3	1	1	Lp(a)
8	m	57	1.6	5	1	until 2005	80.5	1.6	0	1	Lp(a)
9	m	63	8.4	5	1	until 1990	72	12.4	1	0	Lp(a)
	6 m/3f	68.5	4.2	5	9	3	83.22	4.0	5	5	9 + 1

Each patient provided written consent for treatment and use of clinical data for anonymous scientific use and publication. The study and patient information have been approved by our local ethics committee (Aerztekammer Niedersachsen No. 30/17/2023).

### 2.2 Hemodialysis

HD was performed using Nikkiso DBB07 with the Nikkiso standard tube set and a highflux dialyzer (Fresenius fx60 or Polyflux 170H). Dialysate flow was 500 mL/min, and dialysate has been composed according to the medical needs of the respective patient. All patients received HD three times per week via AV‐fistula (BC‐Shunt or cubital‐shunt). The extracorporeal volume of the HD system amounts to approximately 300 mL. The median time on dialysis was 5.0 h (4.0–5.5 h).

### 2.3 LA

Standard selective LA procedures used during this study have been described in the literature [14], and were performed according to manufacturers' instructions. Double‐filtration plasmApheresis (Monet, Fresenius Medical Care, Bad Homburg, Germany) and whole blood (Liposorber DL systems; Kaneka, Osaka, Japan), or ApoB100 immunoadsorption (TheraSorbLDL, Miltenyi Biotec, Bergisch Gladbach, Germany) were used.

**FIGURE 1:**
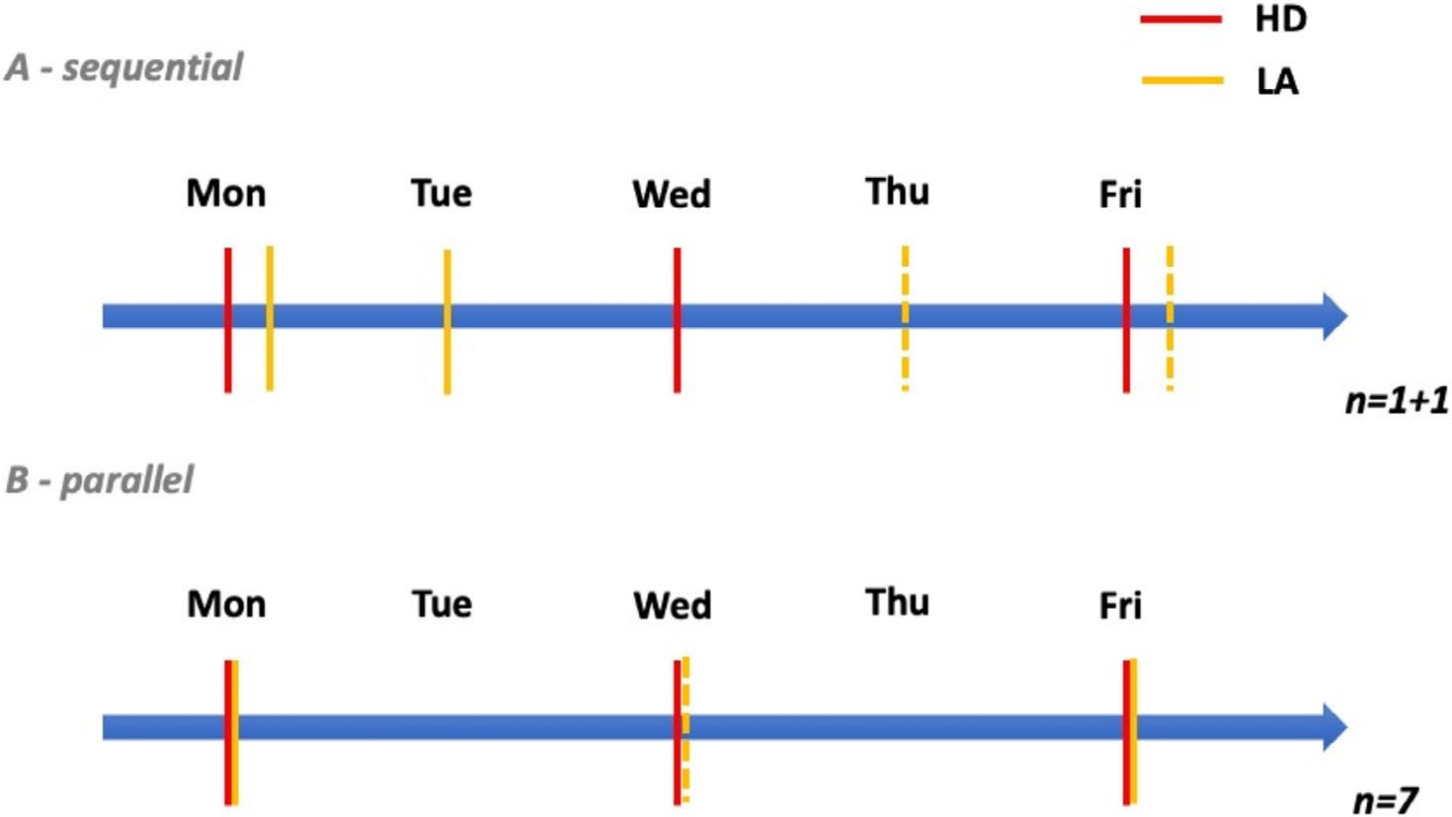
Treatment protocols of combined hemodialysis (HD) and Lipoprotein Apheresis (LA) treatment. All patients were treated thrice weekly by HD (red lines) and one (orange line) to two times weekly (dotted orange line indicates alternative or second treatment per week) by LA. (A) Two of nine patients were treated sequentially with one patient having HD and LA consecutively on the same day and one patient having HD 1 day and LA the next day. (B) All other patients were treated in parallel with HD and LA at the same time. In case of LA once weekly, LA and HD were combined on Wednesday. In case of two LA treatments per week, HD and LA were conducted on Monday and Friday.

**FIGURE 2:**
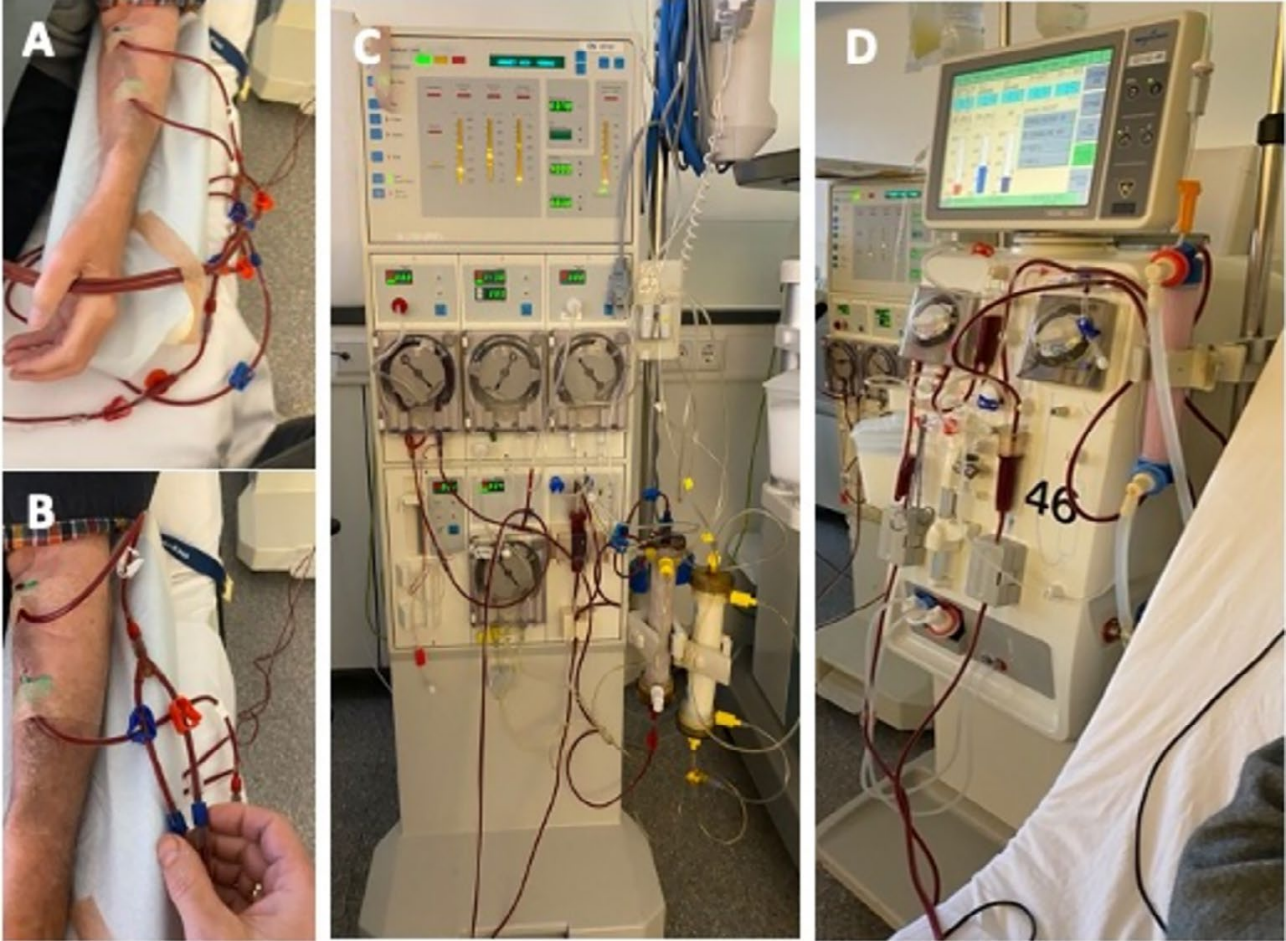
Example of combined hemodialysis (HD) and lipoprotein apheresis (LA) treatment. 15G needles are placed in the forearm fistula of the patient (A). Blood flow is split up by a Y‐piece (B) leading one part of the blood to the LA machine (C, Doublefiltration Fresenius MONET) and one part to the hemodialysis machine (D, Nikkiso DBB07). In parallel, back flow of the blood is conducted via another Y‐piece, and the cleared blood goes back to the patient (A, B).

LA treatment was performed either (sub‐) sequentially (Figure 1A) to HD, immediately after HD (*n* = 1) or the day after HD (*n* = 1), or in parallel to HD (*n* = 7) (Figure 1B). Parallel treatment was performed using 2 needles connected with a Y‐tube, splitting blood flow between HD and LA machines (Figure 2A,B).

For sequential HD‐LA treatment, the whole blood Kaneka Liposorber DL‐Systems was utilized. For parallel LA (*n* = 7) the TheraSorb‐Life18, Life21 (Miltenyi Biotec) Apheresis‐unit or the Fresenius Membrane Filtration Optimized Novel Extracorporeal Treatment (MONET)‐System was utilized, specifically chosen for their relatively small extracorporeal volume compared to other LA‐Systems in order to reduce the total extracorporeal treatment. The Miltenyi TheraSorb system (Miltenyi Biotec) uses two adsorbers installed in parallel (the 200 mL variant was used for the LDL adsorber) that remove APOB100 containing Lipoproteins, such as LDL, Lp(a), and VLDL from the patient's plasma. Plasma volumes processed during LA treatments were adjusted to the patient's individual need. Both adsorbers are produced for multi‐use (Up to 20 treatments) and were stored in a specific preservation solution at 2°C–8°C; after approximately 20 LA treatments, the adsorbers were replaced.

The MONET‐System separates the plasma from whole blood using a plasma filter, and subsequently LDL and Lp(a) are removed by the MONET‐filter. Afterwards, the cleared plasma is re‐merged with the remaining blood and returned to the patient. The extracorporeal volume of the different LA systems amounts to approximately 150 to 200 mL per treatment.

For parallel HD‐LA treatment, LA was started approximately 15 min after HD was initiated (in order to reduce potential episodes of hypertension). LA treatment was continued until the planned dialysis time was completed. Thus, dialysis time indirectly determines treatment volumes of LA.

### 2.4 Anticoagulation

Anticoagulation for all treatments (LA and HD) was achieved using unfractionated heparin.

### 2.5 Primary Outcome Measures

Due to the small sample size, adverse cardiovascular events (ACVE) were the primary composite outcome parameter including MACE as defined by Jaeger et al. [19] and Leebman et al. [16]. This includes cardiovascular death, coronary bypass surgery, nonfatal myocardial infarction, percutaneous coronary intervention or stent, as well as all documented cardiovascular events in all vascular beds. Additionally, MACE includes non‐hemorrhagic cerebrovascular events such as prolonged reversible ischemic neurologic deficit (PRIND), transient ischemic attack (TIA), ischemic stroke, carotid percutaneous transluminal angioplasty/surgery, peripheral vascular events of lower extremities or renal arteries or angioplasty/surgery in that area, or venous thrombotic events (i.e., pulmonary embolism or deep venous thrombosis).

### 2.6 Secondary Outcome Measures

Baseline lipid levels and renal parameters were determined at 2 years before initiation of LA (Table 2) including LDL‐C, Lp(a), triglycerides (TG), s‐creatinine, hemoglobin, urinary albumin‐creatinine‐ratio (uACR), PO4, HbA1c, and kt/V (if applicable). The parameter Kt/V is a measurement of the efficacy of a hemodialysis session. It identifies the effective removal of a specific solute (clearance K, usually blood‐urea) resulting from a given treatment (hemodialysis, characterized by time t) in a given patient (with a specific volume of distribution V for the solute considered).

**TABLE 2:** Risk profile before initiation of LA.

	Lp(a) (nmol/L)	LDL‐C (mg/dL)	TG (mg/dL)	KT/V	PO4 (mg/dL)	HbA1c (%)	uACR (mg/g)	S‐creatinine (mg/dL)
Mean (Lp(a) median)	242.67	96.01	229.5	1.64 (*n* = 2)	1.42	6.47	1456	4.93
SD (Lp(a) range)	164–400.1	±44.25	±129.6	±0.28	±0.15	±1.11	±1559	±3.0

For HD efficacy kt/V, hemoglobin, uACR, time per dialysis session, and serum creatinine were determined at least once every 3 months. For LA efficacy, absolute concentrations of LDL‐C, Lp(a) and TG and reduction rates of LDL‐C and Lp(a) were determined.

### 2.7 Method of Lp(a) Measurement

Lp(a) is measured externally by our cooperating laboratory (https://www.labor‐limbach‐lehrte.de/olvz/3673/details/5244) by using the Roche Cobas C Tina‐Quant Lp(a) Gen.2 test system, which is based on a latex‐enhanced immunoturbidimetric assay. The results are given in nmol/L.

### 2.8 Statistics

If applicable, statistical comparison of groups was performed using paired *t*‐tests for comparison between the different time points of the group using Microsoft Excel for Mac 2019, Version 16.65.

## 3 Results

### 3.1 Patients

Patient characteristics are summarized in Table 1. In total, *n* = 9 patients, 6 male and 3 female, were analyzed for our study. As of the 1st of March 2021, the median age (range) was 68.5 (57–80) years, the mean dry‐weight (±SD) was 83.22 ± 18.36 kg. Median (range) years on HD were 4.2 (1.5 to 23.6) years and median years on LA were 4.0 (1.6 to 12.4) years. All patients suffered from medically compensated arterial hypertension; three patients had a smoking history, but all three stopped smoking many years ago, and five had diabetes mellitus type 2. The mean HbA1c of all patients at baseline was 6.47% ± 1.11% and went down to 5.99% ± 1.09% under combined treatment.

Of note, the indication for LA of all patients was hyperlipoproteinemia(a) and in 1 patient, additional hypercholesterolemia. Family history for cardiovascular events was positive in all patients (not shown).

Of *n* = 9 patients, five started LA prior to HD; in the other *n* = 4, vice versa (Table 1).

The treatment frequency for LA was 1–2 treatments/week with a mean of 1.61, and for HD, three treatments per week with a mean time per treatment of 4.94 ± 0.42 h.

### 3.2 Baseline and Treatment Levels of Lipid Parameters

Baseline (3 years prior to initiation of LA) parameters are summarized in Table 2 and levels of the same parameters under LA treatment are displayed in Table 3.

**TABLE 3:** Risk profile 3 years after initiation of LA.

	Lp(a) (nmol/L)	LDL‐C (mg/dL)	TG (mg/dL)	KT/V	PO4 (mg/dL)	HbA1c (%)	uACR (mg/g)	S‐creatinine (mg/dL)	RR Lp(a) (%)	RR LDL‐C (mg/dL) (%)
Mean (Lp(a) median)	136.31	72.82	221.4	1.38	1.61	5.99	423	7.03	64.15	57.26
SD (Lp(a) range)	121.73–232.04	±18.33	±141.6	±0.15	±0.26	±1.09	±812	±2.12	±5.45	±7.93

The initial median value (±range) of Lp(a) significantly decreased under LA treatment from 242.67 (±164.0–400.10) to 136.32 (±129.46–223.04) nmol/L (*p* < 0.001) and the mean value (±SD) of LDL‐C (all patients receiving the maximum tolerated dose of statins at any time point of study (Table 1)) decreased from 96.01 ± 44.25 to 72.82 ± 18.33 mg/dL (*p* = 0.113), respectively (Tables 2 and 3) and was further lowered by LA with acute reduction rates (RR), comparing pre‐and post‐LA levels, for Lp(a) and LDL‐C of an additional 64.15 ± 5.45 and 57.26% ± 7.93%, respectively. This results in median Lp(a)‐levels of approx. 48.9 nmol/L and mean LDLlevels of approx. 32 mg/dL immediately after treatment. Thus, LA strongly reduces the levels of atherogenic lipids.

### 3.3 Baseline and Treatment Levels of Renal Parameters

Three years prior to the initiation of LA (baseline) only *n* = 2 of the nine patients were on dialysis (mean kt/V 1.69 ± 0.28, Table 2); the rest suffered from chronic kidney disease stage G3b to G5. Taken together, the mean s‐creatinine was 4.93 ± 3.0 mg/dL and the mean uACR was 1.45 ± 1.55 g/g (Table 2), which seemed to be relatively high due to *n* = 2 patients suffering from nephrotic proteinuria of more than 4 g/g creatinine, which would have further increased LDL‐C, TG, and Lp(a) levels in these two respective patients.

At 1st of March 2021, all patients underwent HD‐treatment with a mean kt/V of 1.38 ± 0.15 (Table 3) suggesting an adequate dose of dialysis in all patients.

Parameters of chronic kidney disease‐mineral bone disorder (PO4 (Tables 2, 3), Calcium, s‐PTH) were compensated during the whole observation period.

### 3.4 ACVE In the Study Population

Occurrence of ACVE in the nine patients was counted yearly (1st of March to 28th of February) according to the complete medical recordings as described above.

**FIGURE 3:**
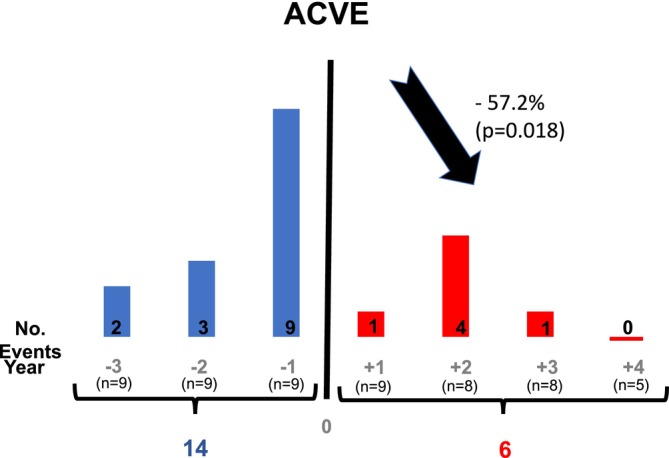
Cumulated number of adverse cardiac or vascular events (ACVE) per year before and after initiation of lipoprotein apheresis (LA). In the 3 years before initiation of LA in total of 14 ACVE occurred in nine patients, whereas in the 4 years after initiation of LA, only six ACVE were recorded (*n* = 9 first year, *n* = 8 year 2–3 and *n* = 5 in the 4th year). This difference in ACVE before and after initiation of LA is statistically significant (*p* = 0.018) suggesting the efficacy of LA treatment in reducing ACVE in hemodialysis patients.

Numbers of ACVE were added in the 3 years prior to the initiation of LA (Figure 3, blue bars) and compared to the numbers of ACVE in the 4 years after the initiation of LA (Figure 3, red bars). Before LA treatment, 14 ACVE occurred in all patients (Figure 3). Thus, 0.52 events per patient and year. In the 4 years under LA therapy, with strongly reduced levels of LDL‐C and Lp(a) (Table 3), in total only six ACVE occurred (Figure 3, red bars), which translates into a significantly reduced frequency of ACVE of only 0.17 events per patient and year (*p* = 0.018). In particular, in the *n* = 5 patients with more than 4 years on LA, not a single ACVE occurred.

### 3.5 Side Effects of Treatment

There were no documented adverse side effects from combination HA and LA treatment in this study. Side effects of LA are usually very rare [15, 16]. However, for the procedure of LA supplementation, a volume (usually sodium chloride 0.9%) is required and leads to an additional volume load for the HD patients of approx. 1000 mL per LAtreatment, which has been taken into account for the calculation of the ultrafiltration rate of the respective patients. In particular, in patients with high interdialytic volume intake, this may result in volume overload.

## 4 Discussion

This study is the first report of patients on HD additionally treated with LA in patients with progressive cardiovascular disease due to hyperlipoproteinemia(a). In these patients, LA treatment proved to be a safe and effective method of reducing plasma lipid levels and the risk of ACVEs.

In chronic kidney disease, including hemodialysis, the role of Lp(a) remains less clear. Patients with chronic kidney disease do have a very high risk for cardiovascular disease in part due to a pro‐atherogenic dyslipidemia including high Lp(a)‐levels [8], whereby the particularly dangerous small and dense Lp(a) molecules are described to be independent of renal function [8] and are increasing the risk for cardiovascular events in HD patients [11]. In our study, we did not determine the number of Kringle IV‐repeats or types of Lp(a), but as the median baseline level of Lp(a) in our group of patients was extremely high at 242.67 nmol/L (approx. 150 mg/dL) representing the > 95th percentile of Lp(a) levels in the population [9], it is very likely that all our patients do have a low number of Kringle IV Type 2 repeats (< 22), which are associated with high concentrations of Lp(a) and increased cardiovascular risk [20]. These Lp(a) levels are considered to be extreme and are, independent of other risk factors, associated with a three‐ to four‐fold risk of myocardial infarction [9]. Focusing on the CKD population, hyperlipoproteinemia(a) seems to be dramatically underestimated and undertreated in this highly vulnerable population. Accordingly, in a sub‐analysis of the CHOICE study, Longenecker et al. [11] found *n* = 833 hemodialysis patients potentially eligible for Lp(a) Apheresis.

There is currently no established pharmacological approach that selectively reduces circulating Lp(a); however, pharmacotherapies such as PCSK‐9‐inhibitors and the interfering mRNA medication Inclisiran are able to lower Lp(a) by almost 30% [21, 22]. For example, in the outcomes trial with Evolocumab, the FOURIER (Further Cardiovascular Outcomes Research with PCSK9 Inhibition in Subjects with Elevated Risk) study, median Lp(a) levels of 37 nmol/L were reduced by a median of 11 nmol/L (27%) [22]. Concerning the risk of experiencing MACE, patients with higher Lp(a) concentrations at baseline were those who derived a greater reduction in the risk of MACE. In particular, a 25 nmol/L reduction in Lp(a) corresponded to a 15% decrement in the relative risk [22]. In the ORION studies, *Inclisiran* was shown to reduce Lp(a) levels from 15.99% to 25.8% [23]. However, these medications are not approved for the treatment of isolated hyperlipoproteinemia(a). More targeted therapies to lower Lp(a) are in development but not yet available [24]. Thus, to date, LA still remains the only established therapeutic intervention. The largest prospective study so far on the effect of Lp(a)‐Apheresis on cardiovascular outcomes in patients with progressive cardiovascular disease and hyperlipoproteinemia(a) is the Pro(a)life study [16, 18] showing pronounced long‐term efficacy in the reduction of cardiovascular events. However, Pro(a)life included only four patients with a GFR < 30 mL/min (of which were three patients with a GFR < 15 mL/min). The RR of cardiovascular events in our study seems to be comparable to the results of the Pro(a)life study, but we described this effect for the first time in hemodialysis patients.

In the current literature, only two reports of combined HD‐LA treatment are available [25, 26] The two studies from Japan both included only patients on LA due to Hypercholesterolemia, but not hyperlipoproteinemia(a) and a cascadeplasma filtration LAstem (Kaneka) was used. Both focused on peripheral arterial disease (PAD): In a first study, 25 patients were enrolled, and the responses of 19 patients to LA were analyzed, of which only 10 patients responded with improvements in ankle‐brachial pressure index (ABI) after treatment, while *n* = 9 were described to be non‐responders with worsened ABI [25]. In the second study, perfusion and microcirculation were examined in PAD after 10 LA treatments compared to baseline. In *n* = 13 patients, Fontaine classification improved (responders), whereas *n* = 5 were non responding, and in *n* = 2, PAD worsened [26]. Independent of the individual response, LA in HD dramatically reduced the burden of atherogenic lipids (LDL‐C, oxLDL, Lp(a), TG) and thrombogenic proteins, such as Lp(a) and fibrinogen in these patients [25, 26]. Thus, LA in HD patients potentially may additionally reduce cardiovascular risk, and our report provides a first hint that Lp(a)‐Apheresis is applicable in hemodialysis patients with extremely high Lp(a) levels and progressive cardiovascular disease.

## 5 Conclusion

A great deal of research is urgently needed to elucidate the relationship between putatively atherogenic lipoproteins and clinical outcomes in advanced CKD. Targeting these lipoproteins may be important to decrease CVD in this population. The results of our study are clearly limited by the small study size and retrospective approach of the analysis.

## Author Contributions

**Tilmann Röseler:** investigation, conceptualization. **Daniel Kayser:** investigation, software, methodology. **Georg Schlieper:** investigation, validation, writing – review and editing. **Wanja M. Bernhardt:** investigation, data curation, formal analysis, writing – original draft.

## Ethics Statement

This study was conducted in accordance with the Declaration of Helsinki (1964) and the responsible ethics committee lower saxony (https://www.aekn.de/aekn/kommissionen/ethikkommission) has approved the study.

## Consent

Patients provided written consent for treatment and use of clinical data for anonymous scientific use and publication. The study and patient information has been approved by our local ethics committee (Medical association of lower saxony No. 30/17/2023).

## Conflicts of Interest

The authors declare no conflicts of interest.

## Data Availability

The data that support the findings of this study are available on request from the corresponding author. The data are not publicly available due to privacy or ethical restrictions.
